# Congenital asymptomatic diaphragmatic hernias in adults: a case series

**DOI:** 10.1186/1752-1947-7-125

**Published:** 2013-05-13

**Authors:** Enrica Bianchi, Paola Mancini, Stefania De Vito, Elena Pompili, Samanta Taurone, Isabella Guerrisi, Antonino Guerrisi, Vito D’Andrea, Vito Cantisani, Marco Artico

**Affiliations:** 1 Department of Sensory Organs, University ‘Sapienza’, Rome, Italy; 2 Department of Radiology, ‘S. Giovanni Calibita’ Isola Tiberina, Fatebenefratelli Hospital, Rome, Italy; 3 Department of Anatomical, Histological, Forensic and Locomotor System Sciences, V. A. Borelli 50, Rome, 00161, Italy; 4 Department of Radiological Sciences, University of Rome Sapienza, Rome, 00159, Italy; 5 Department of Radiology, Oncology and Pathology, University ‘La Sapienza’, Policlinico Umberto I, Rome, Italy

**Keywords:** Congenital diaphragmatic hernia, CT scan, Diaphragmatic malformation, Plain X-ray films, Surgery

## Abstract

**Introduction:**

Congenital diaphragmatic hernia is a major malformation occasionally found in newborns and babies. Congenital diaphragmatic hernia is defined by the presence of an orifice in the diaphragm, more often to the left and posterolateral, that permits the herniation of abdominal contents into the thorax. The aim of this case series is to provide information on the presentation, diagnosis and outcome of three patients with late-presenting congenital diaphragmatic hernias. The diagnosis of congenital diaphragmatic hernia is based on clinical investigation and is confirmed by plain X-ray films and computed tomography scans.

**Case presentations:**

In the present report three cases of asymptomatic abdominal viscera herniation within the thorax are described. The first case concerns herniation of some loops of the large intestine into the left hemi-thorax in a 75-year-old Caucasian Italian woman. The second case concerns a rare type of herniation in the right side of the thorax of the right kidney with a part of the liver parenchyma in a 57-year-old Caucasian Italian woman. The third case concerns herniation of the stomach and bowel into the left side of the chest with compression of the left lung in a 32-year-old Caucasian Italian man. This type of hernia may appear later in life, because of concomitant respiratory or gastrointestinal disease, or it may be an incidental finding in asymptomatic adults, such as in the three cases featured here.

**Conclusions:**

Patients who present with late diaphragmatic hernias complain of a wide variety of symptoms, and diagnosis may be difficult. Additional investigation and research appear necessary to better explain the development and progression of this type of disease.

## Introduction

Congenital diaphragmatic hernia (CDH) is an idiopathic human malformation that usually presents in the newborn period. CDH is a relatively common condition that occurs in less than one to five babies per 1000 births [1]. It seems to be slightly more frequent in men and less frequent in Blacks [2,3]. This disease may be detected during fetal life when screening ultrasonography demonstrates herniation of the bowel or liver into the thorax and permits a correct pre-natal diagnosis in 50 percent to 90 percent of cases. Polyhydramnios may lead to antenatal diagnosis in some severe cases [4]. The onset of CDH is generally due to respiratory distress or gastrointestinal disease, because of obstruction and incarceration of herniated bowel loops. Respiratory insufficiency is often secondary to pulmonary hypoplasia and persistent pulmonary hypertension. The cause of pulmonary anomalies is discussed in the literature [5,6] and seems to be caused by intra-thoracic compression of the lungs by the herniated abdominal viscera, leading to alterations of pulmonary blood flow. Pulmonary hypoplasia in CDH is not just the result of direct compression of lung parenchyma, because pulmonary development is disrupted early in gestation before visceral herniation has occurred. However, the etiology of pulmonary hypoplasia in CDH remains unknown. Respiratory and cardiovascular functions are severely compromised at birth and this, together with the frequently associated malformations, cause considerable rates of mortality and morbidity. CDH should be included in the differential diagnosis with pneumonia and other respiratory or gastrointestinal diseases [7,8]. The neonatal symptoms of CDH are heralded by respiratory distress with insufficient oxygenation, excavated abdomen with sternal protrusion and displacement of the heart sounds to the contralateral side. The symptoms of insufficient gas exchange are associated with those of persistent pulmonary hypertension [9,10] caused by arteriolar constriction and closure of the pulmonary arterial bed that forces maintenance of a pattern of persistent fetal circulation in which the blood from the right ventricle is shunted to the left heart, preventing effective gas exchange. The manifestations of CDH may occur at any age as mild respiratory distress or may even be an unexpected finding during a medical check-up for other reasons [11]. In these cases, a hernial sac is most often present [12]. Other organs may be involved in CDH [13] because associated malformations are frequent [14].

The heart and vessels are often abnormal in patients with CDH. A diagnosis of CDH is unexpected if the patient is asymptomatic. In such cases radiological findings play a predominant role. The radiological features on X-ray films are not always easy to detect. Typically, radiological images show intra-thoracic gas-filled loops of the bowel with a contralateral shift of the mediastinum. In some cases, the herniated bowel loops may be complicated, such as in obstruction or volvulus, and the fluid-filled bowel loops appear as an opacity mimicking a lung consolidation. In other cases the gas-filled bowel loops may simulate a pneumothorax [15,16].

A computed tomography (CT) scan is the radiological investigation that allows the highest accuracy for a correct diagnosis. It provides a precise assessment of the anatomical relationships between the viscera, and congenital malformations, as in our cases.

Very few cases of delayed presentation of CDH in asymptomatic adults are described in the literature. We report three cases in adult patients, presenting with herniation of the abdominal viscera in the thorax, who underwent CT scans for symptoms not related to either herniation or respiratory disease. In one of these cases, a rare occurrence of right abdomen viscera herniated into the right hemi-thorax was observed.

## Case presentations

### Case 1

**Figure 1:**
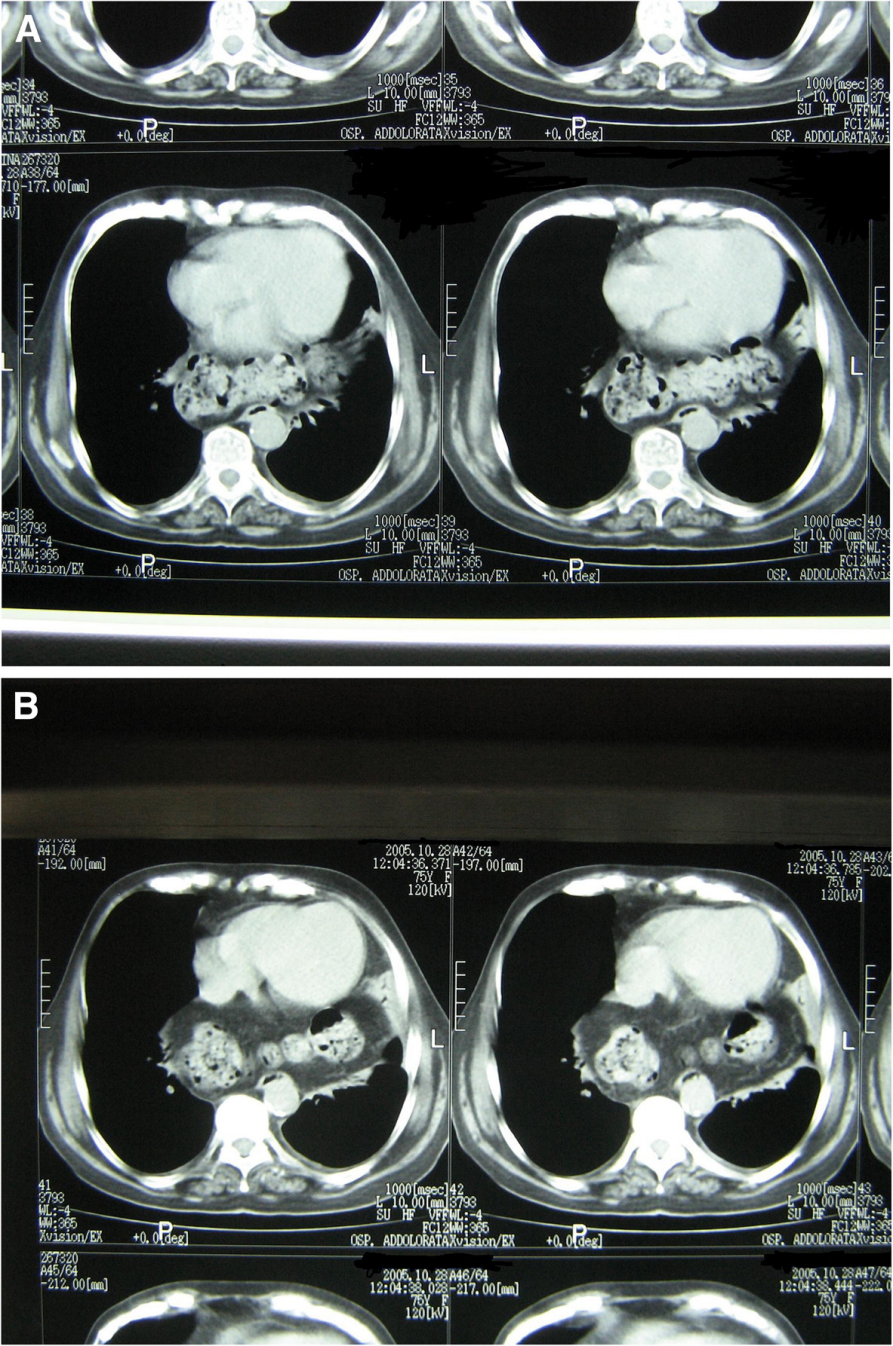
**(A,B) Axial CT scan of the chest.** Gas-filled large bowel loops are visible behind the heart, laying anteriorly to the spine and the aorta, with part of the abdominal fat.

A 75-year-old Caucasian Italian woman with hypereosinophilia underwent a CT scan to evaluate her lung parenchyma. The eosinophilia was diagnosed as idiopathic. No respiratory symptoms were reported by our patient, or any symptoms related to gastrointestinal disease. Our patient agreed to a CT scan after signing the informed consent. Our patient underwent a CT scan without intravenous contrast administration. The mediastinum window level showed a small pleural fluid collection on the left side of the thorax and a few small lymph nodes around the trachea (Figure 1). No focal or diffuse lung parenchyma pathological involvement was found. The esophagus was correctly positioned on the right side of the descending aorta, besides the trachea. Posteriorly to the tracheal bifurcation, gas-filled large bowel loops were evident (Figure 1), located in the posterior mediastinum, anterior to the esophagus and the descending aorta. The bowel loops occupied the left inferior hemi-thorax, near the left heart ventricle. The other abdominal viscera, such as the spleen, stomach and the rest of the bowel loops, were situated in their correct anatomical positions. Our patient did not receive surgical treatment because she was asymptomatic.

### Case 2

**Figure 2:**
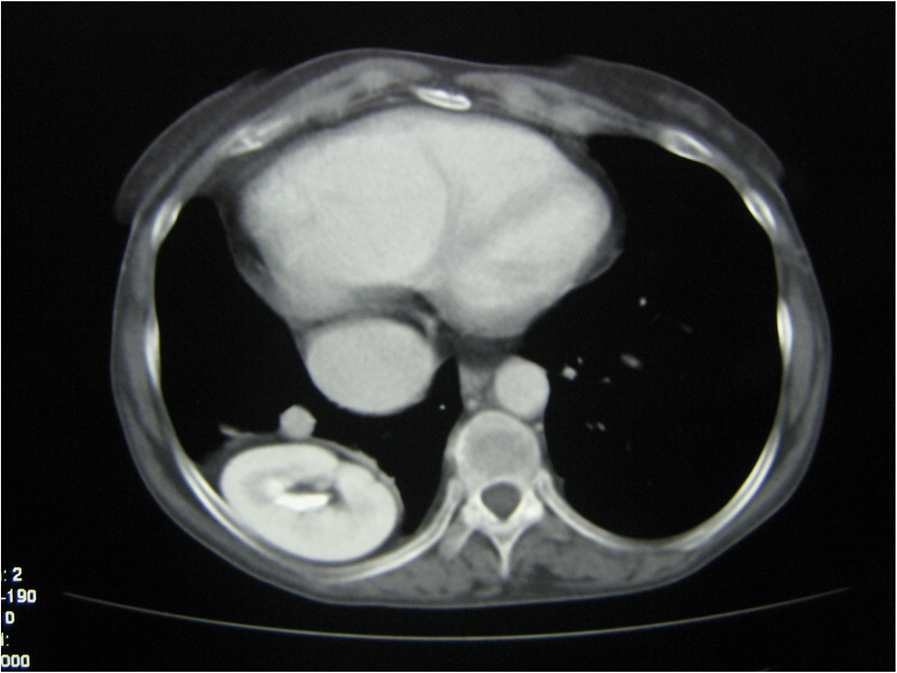
**Computed tomography scan.** Part of the right kidney and an enlarged inferior vena cava are shown, flowing into the also enlarged right atrium.

A 57-year-old Caucasian Italian woman with right heart overflow underwent a CT scan with intravenous contrast administration. Our patient agreed to a CT scan after signing the informed consent. The mediastinum window level was performed in the venous phase after the injection of contrast medium. The CT showed some anomalies of the vascular structures: the inferior vena cava was enlarged from its retro-hepatic segment as far as the efflux in the right atrium, which also appeared mildly enlarged (Figure 2). The pulmonary vessels of the right hemi-thorax, as well as the right pulmonary artery, appeared to be increased in diameter, as in a blood overflow condition. The left pulmonary vessels were normal. CT scans showed the superior portion of the right kidney and a part of the liver parenchyma (which may be ascribed to the VI hepatic segment) occupying the right basal hemi-thorax. The middle and inferior part of the kidney remained in the abdomen, with the right adrenal gland and the rest of the renal pelvis (Figure 2). The right renal parenchyma appeared slightly larger than the contralateral one, but the renal pelvis and the ureter were normal. Renal function appeared regular, with normal elimination of the contrast agent bilaterally. The left kidney was in a slightly higher position in the abdomen, contiguous to the spleen, in the abdominal cavity. Surgical repair with a tension-free patch of Gore-Tex was proposed to our patient, but she refused surgery because of the possible post-operative risks and complications; she was subsequently discharged.

### Case 3

**Figure 3:**
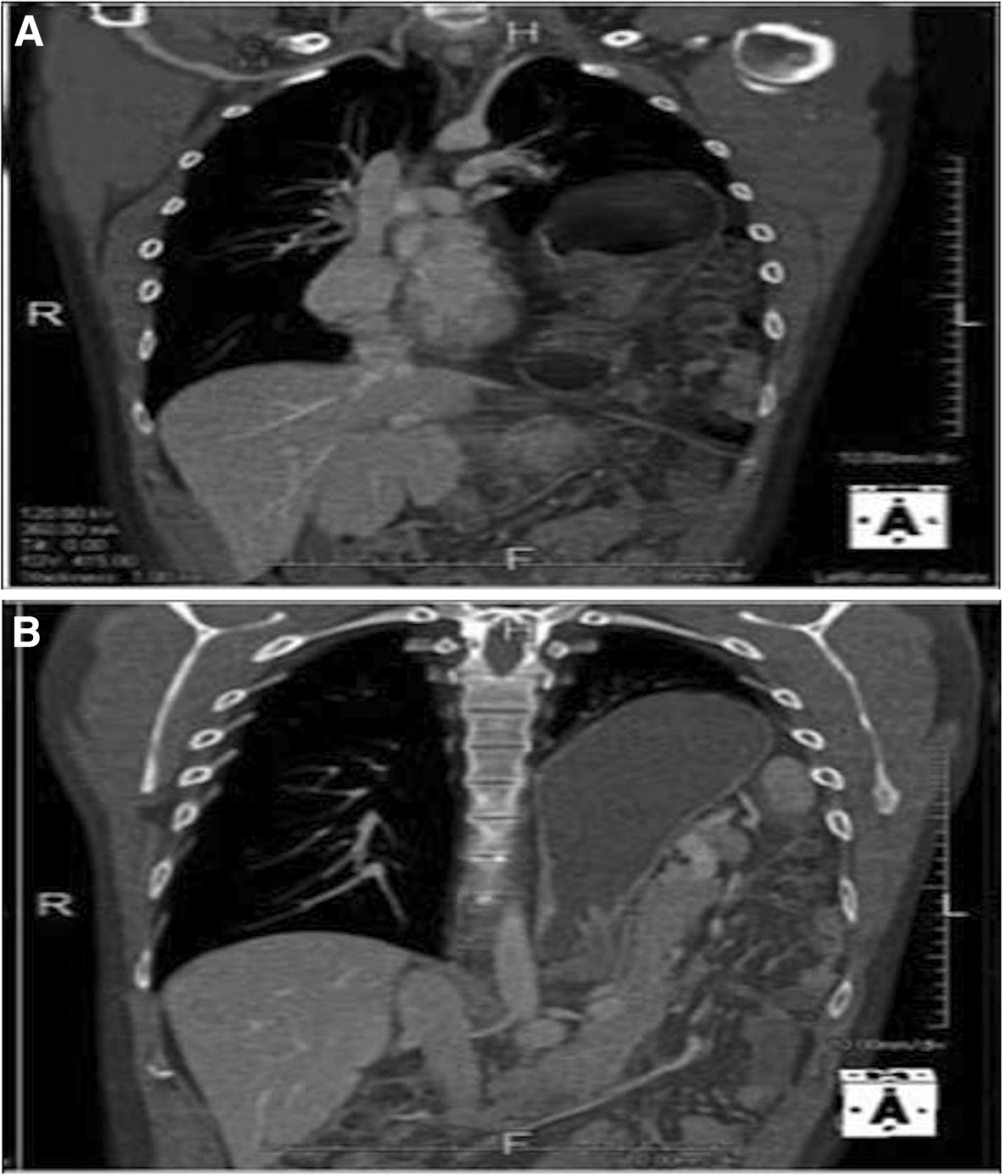
**(A,B) Computed tomography scan of the chest.** Coronal reconstruction. Part of the stomach, adjacent to the heart (**A**), is clearly visible. Some bowel loops (**B**) are also visible on left side of the chest: the left lung is displaced and compressed.

A 32-year-old Caucasian Italian man with hypereosinophilia underwent a CT scan to evaluate his lung parenchyma. The eosinophilia was diagnosed as idiopathic. Our patient complained of occasional vomiting, nausea, epigastric pain and palpitations. Respiratory sounds were found to be diminished at the left basal region on auscultation, where occasional peristaltic sounds were appreciable. Our patient agreed to a CT scan after signing the informed consent. Our patient underwent a CT scan without intravenous contrast administration (Figure 3A,B), which demonstrated herniation of the stomach (adjacent to the heart, Figure 3A) and of bowel into the left side of the chest with compression of the left lung (Figure 3B). Surgical repair with a tension-free patch of Gore-Tex was proposed to our patient, but he refused surgery because of his scarce and intermittent symptoms and was subsequently discharged.

## Discussion

The diaphragm divides the thorax from the abdomen and is derived from several sources during embryological development, mainly the septum transversum. This is situated at first rostrally to the somites in the embryonic period and acts as a mesodermal bridge between the pericardial and the umbilical vesicle cavities. Its cranial portion becomes covered by pericardium and pleura, whereas its ventral part is a sagittal mesentery that contains the expanding liver. The septum transversum gives rise to the majority of the diaphragm and descends from the cervical to thoracic level during embryonic development. Normal diaphragm closure occurs at the eighth week.

The other parts of the diaphragm consist of the pleuroperitoneal membranes and the mesenchyma of the body wall. The pleuroperitoneal canals may be closed by the contribution of the subjacent organs, such as the liver and the suprarenal glands. The skeletal muscle of the diaphragm is probably derived *in situ* from the body wall and also through the migration from cervical myotomes.

The CDH is a displacement of the abdominal organs into the thoracic cavity through a weak area or a distinct defect in the diaphragm. The causes of precipitating viscera herniation may be related to mechanical or pressure changes in the thoracoabdominal cavities. The most frequent types of diaphragmatic hernia are the left posterolateral (Bochdalek hernia) and the sternocostal (Morgagni hernia) types. A Bochdalek hernia, resulting from inadequate closure of the posterolateral pleuroperitoneal membrane, is the most frequently seen congenital diaphragmatic hernia. Defects occur more frequently on the left side than on the right side of the diaphragm, and the abdominal contents, including stomach, bowel loops, liver, spleen or fat tissues, may be displaced into the thoracic cavity. A posterolateral hernia on the right side is very rare and this is probably attributable to the protection provided by the liver. Foramen of Morgagni hernias are rare diaphragmatic hernias, usually occurring on the right and located in the anterior mediastinum because of the retrosternal location of the foramen of Morgagni, described as an anterior diaphragmatic defect. In adults, foramen of Morgagni hernia is also associated with obesity, trauma, weight lifting, or other causes of increased intra-abdominal pressure.

The most frequent cause of herniation of the abdominal viscera in adults seems to be trauma, whereas in babies or newborns it is most often attributable to congenital absence or defective fusion of the septum transversum or the pleuroperitoneal membrane. The detection of diaphragmatic hernia is made with pre-natal ultrasonography in 50 percent to 90 percent of cases [17,18]. The intestine and the liver may be in the thorax and the lungs are small. Ultrasound scans allow detailed assessment of the heart. Lung growth is measured as a proportion of head growth. The lung-to-head ratio (LHR) has some prognostic value [19-25], because when it is below 1, survival is compromised [26]. When diagnosis is made *in utero*, amniocentesis is often performed for detecting chromosomal aberrations [27] and may help to estimate lung maturity [28]. After birth, a diagnosis can readily be made on the basis of symptoms and physical signs. A plain X-ray of the thorax and abdomen provides details of the position of the herniated viscera. Blood gases and pH status reflect the efficiency of gas exchange. A physical examination may be sufficient, but passing a naso-gastric catheter into the stomach before a plain X-ray of the thorax and abdomen may help to locate it or to detect any esophageal displacement [29]. In some rare cases, herniation of viscera through the diaphragm is an incidental finding in adult patients undergoing plain X-ray films or CT scans for other symptoms not related to this pathology. Since our three adult patients had asymptomatic or poorly symptomatic diaphragmatic hernia we did not refer the herniation to any traumatic event and so the diaphragmatic defects were, presumably, congenital. A large proportion of fetuses with CDH are diagnosed *in utero* and termination of gestation is sometimes preferred, particularly when chromosomal aberrations and syndromes are present [30-33]. The possibility of fetal instrumentation directed to alleviate the consequences of the herniation is becoming a progressively more acceptable alternative. The current development of minimally invasive surgery has brought about the immediate effect of making fetendoscopic balloon tracheal occlusion possible in fetuses with LHR below 1 [34]. Experimental and clinical evidence suggest that biochemical lung maturation is delayed in fetuses with CDH [35] and this led to the assumption that this process is accelerated by administration of maternal corticosteroids as carried out for premature deliveries. However, since the evidence of the benefits of this medication are not totally convincing [36], a multicenter trial could be useful [37].

Whenever pre-natal diagnosis is made, gestation should be prolonged until near term if possible [38] and there is no evidence of the benefits of delivery by Caesarean section [38]. Treatment after birth requires extra-corporeal membrane oxygenation (ECMO) prior to surgical correction. Extra-corporeal membrane oxygenation can be seen as a safety net maintained until proper gas exchange occurs. Cannulation of both the right carotid artery and jugular vein and connection to a circuit with a membrane gas exchange chamber allows oxygenation and CO_2_ disposal without participation of the lung, which is thus preserved from any pressure insult [39]. This technique is probably a good adjunct in a limited number of patients in whom predicted severe lung hypoplasia would make adequate gas exchange impossible or in those in whom reversal to the fetal pattern of circulation becomes unmanageable. In the past, surgical repair of CDH used to be a life-saving emergency procedure. It is presently accepted that this procedure should be undertaken only once cardio-respiratory functions have been stabilized. A policy of delayed surgery coupled with gentle ventilation and occasional ECMO support has yielded the best results to date. The goal of all forms of pre-operative treatment is to obtain ‘stabilization’ of the patient: this requires acceptable oxygenation and CO_2_ disposal with stable pulmonary pressures, tolerable shunting, good myocardial function and adequate renal clearance with reduced or withdrawn inotropic drugs. Under general anesthesia, a subcostal or transverse abdominal incision is made and the herniated viscera are carefully reduced into the abdomen: the diaphragmatic orifice is closed with interrupted sutures without leaving an intercostal tube. A tube was routinely used in the past [40] until it was realized that underwater seals cause increased respiratory work and overdistension of the hypoplastic lung that may reduce ventilation even further. A tubeless policy was then advised [41]. When the defect is too large, a prosthetic patch is used to achieve closure [42]. It is sutured to the rims of the orifice with interrupted sutures and, to avoid excessive tension and enlargement of the hemi-thorax, cone shaping of the patch can be beneficial [43]. The use of a patch seems to increase the risk of re-herniation [44] although it should be acknowledged that patients requiring a patch have larger defects that entail higher morbidity [45]. Abdominal wall or dorsi muscle flaps have also been used for CDH repair [46]. Since the advent of minimally invasive surgery (MIS) thoracoscopic [47] or laparoscopic [48] approaches have been proposed for CDH repair. MIS is a good approach in cases diagnosed during infancy with less severe symptoms. Regardless, some concerns about this particular approach in the newborn period have been expressed on the basis of excessive peri-operative hypercapnia and prolonged post-operative low brain oxygenation. The risk of recurrence after a minimally invasive approach has been found to be similar to that of open surgery.

Congenital diaphragmatic hernias mainly present in the neonatal period and are associated with a mortality that has not changed much despite the advances made in critical care. Rarely, these hernias present later in life, some even in adulthood. There are numerous reports of CDH presenting after infancy. These patients are either asymptomatic or have minimal respiratory symptoms, possibly because the lungs are not hypoplastic.

Late-presenting CDH is often difficult to diagnose, and delays in treatment are common. All diaphragmatic defects could be closed by an abdominal approach without post-operative complications. Clinical symptoms disappeared post-operatively. In adulthood a CDH may present with gastrointestinal tract symptoms that may include intermittent abdominal pain, vomiting, and dysphagia. Respiratory symptoms usually include dyspnea and chest pain [49]. Symptoms may be intermittent or acute depending on the extent of herniation of abdominal viscera into the thorax. An acute presentation is usually due to incarceration, obstruction, or strangulation of the herniated viscera [50]. Diagnosis is ascertained by a combination of chest X-rays, CT and magnetic resonance imaging (MRI), as well as upper gastrointestinal and bowel double-contrast studies. Typical findings on a CT scan would be the abutment of fat or soft tissue along the upper surface of the diaphragm, characteristic posterolateral location on the hemidiaphragm, diaphragmatic discontinuity adjacent to the mass, and continuous density above and below the diaphragm through the defect [51]. Bochdalek hernia may be misdiagnosed as pleural effusion, pneumonia, tension pneumothorax, lung cysts, or atelectasis.

A careful analysis of chest films and a thorough search for connecting bowel segments passing through the diaphragmatic defect may help to avoid incorrect diagnosis and an undesirable delay in treatment. Confusion with pneumonia or pneumothorax can be diminished by placing a feeding tube and instillation of contrast material.

Management of a Bochdalek hernia includes reducing the abdominal contents and repairing the defect through a laparotomy or thoracotomy. Successful laparoscopic and thoracoscopic repairs of Bochdalek hernias have both been described. Right-sided defects are usually dealt with by a thoracic or thoracoabdominal approach because of the presence of the liver. For left-sided hernias some authors suggest a transthoracic approach while others advise a transperitoneal approach [52]. The indications for repair of congenital diaphragmatic hernia are the same as those to treat any hernia, and should take into account the patient’s overall medical condition. The outcome of adult patients suffering from Bochdalek hernia depends on the type of clinical presentation. Delay in the diagnosis of CDH can result in significant morbidity [53].

## Conclusions

The cases reported in our study are delayed presentations of CDH in a posterior location (Bochdalek’s-like viscera herniation) detected as an incidental finding on chest CT scans.

In the first case there was herniation of the large bowel on the posterior side of the mediastinum. The second case appeared to be more unusual because the viscera were herniated through a defect on the right side of the diaphragm in the right hemithorax. Moreover, in the second case the herniated viscera exerted compression on the right side of the heart, resulting in a blood overflow in the pulmonary circulation and a small pleural fluid collection on the left side of the thorax. In the third case, there was herniation of the upper part of the stomach into the thorax through a tear or weakness in the diaphragm. In such cases, where hernia symptoms are severe and chronic acid reflux is involved, surgery is sometimes recommended, since chronic reflux can severely injure the esophagus and even lead to esophageal cancer. Generally speaking, plain X-ray films may be considered the primary radiological investigation able to show the presence of gas-filled bowel loops within the thoracic cavity. However, in our study, our patients initially underwent chest CT scans because this investigation facilitates diagnosis of such anatomical variations and establishes the relationships between the abnormally positioned viscera.

We report three cases of delayed presentation of a potentially life-threatening CDH. The variable clinical features of CDH presenting beyond the neonatal period may result in clinical and radiological misdiagnosis. CDH with complicating mediastinal shift and respiratory distress requires urgent gastrointestinal decompression and respiratory support. The most significant factor in achieving diagnostic success is to consider it early in the differential diagnosis to avoid misguided or delayed therapy.

In conclusion, CDH is a complex condition probably caused by disturbed molecular signaling during organogenesis. The diaphragmatic orifice is invariably accompanied by pulmonary hypoplasia with vascular hyper-reactivity that causes deficient gas exchange and persistent pulmonary hypertension. In addition, other malformations that further complicate the clinical course may be present. Regardless, many aspects of the disease are still unknown and, given that the incidence is relatively high, that the expenses involved in the current treatments are overwhelming and that the sequelae are frequent, more research efforts into causation, prevention and treatment are warranted.

## Consent

Written informed consent was obtained from the patients for publication of this manuscript and any accompanying images. A copy of the written consent is available for review by the Editor-in-Chief of this journal.

## Competing interests

The authors declare that they have no competing interests.

## Authors’ contributions

EB, EP, PM, ST, SDV, IG, AG, VDA and VC analyzed and interpreted the data from our patients regarding radiological investigation by plain X-ray films and CT scans. EB and MA were the main contributors in writing the manuscript. All authors read and approved the final manuscript.
